# Indirect implications of COVID-19 prevention strategies on non-communicable diseases: An Opinion Paper of the European Society of Hypertension Working Group on Hypertension and Cardiovascular Risk Assessment in Subjects Living in or Emigrating from Low Resource Settings

**DOI:** 10.1186/s12916-020-01723-6

**Published:** 2020-08-14

**Authors:** Pietro A. Modesti, Jiguang Wang, Albertino Damasceno, Charles Agyemang, Luc Van Bortel, Alexandre Persu, Dong Zhao, Faical Jarraya, Ilaria Marzotti, Mohamed Bamoshmoosh, Gianfranco Parati, Aletta E. Schutte

**Affiliations:** 1 Department of Experimental and Clinical Medicine, School of Medicine, Universita’ degli Studi di Firenze, Azienda Ospedaliero Universitaria Careggi, Largo Brambilla 3, 50134 Florence, Italy; 2 The Shanghai Institute of Hypertension, Ruijin Hospital, Shanghai Jiaotong University School of Medicine, Shanghai, China; 3 Department of Cardiology, Faculty of Medicine, Eduardo Mondlane University, Maputo, Mozambique; 4 Department of Public & Occupational Health, Amsterdam Public Health (APH) Research Institute, Amsterdam University Medical Centres, University of Amsterdam, Meibergdreef 9, Amsterdam, 1105 AZ the Netherlands; 5 Division of Clinical Pharmacology, Department of Basic and Applied Medical Sciences, Ghent University, Ghent, Belgium; 6 Division of Cardiology, Cliniques Universitaires Saint-Luc and Pole of Cardiovascular Research, Institut de Recherche Expérimentale et Clinique, Université catholique de Louvain, Brussels, Belgium; 7 Department of Epidemiology, Beijing Anzhen Hospital, Capital Medical University, Beijing, China; 8 Université de Sfax, Laboratoire de Recherche LR19ES11 (Ex. UR12ES14), Faculté de Médecine, Avenue M. Boulila, Sfax, 3029 Tunisie; 9 University of Science and Technology, Sana’a, Yemen; 10 Department of Medicine and Surgery, University of Milano-Bicocca, Department of Cardiology, S. Luca Hospital, IRCCS Istituto Auxologico Medicine and Surgery, Milan, Italy; 11 Hypertension in Africa Research Team, North-West University, Potchefstroom, South Africa; 12 School of Public Health & Community Medicine, Faculty of Medicine, University of New South Wales and the George Institute for Global Health, Sydney, Australia

**Keywords:** Coronavirus, Non-communicable diseases, Hypertension, Diabetes, Cardiovascular disease, Cancer, Respiratory disease, SARS-CoV-2, COVID-19

## Abstract

**Background:**

After its outbreak in China, the novel COronaVIrus Disease 19 is spreading across the globe. It is an emergency the world has never seen before.

**Main text:**

The attention of health systems is mainly focused on COronaVIrus Disease 19 patients and on the risk that intensive care units might be overwhelmed by the serious pulmonary complications. Different countries are also attempting to establish infection prevention and control strategies which proved effective in China where the outbreak was initially reported. We reflect on important lessons to be learnt from different countries. The effects that infection prevention and control strategies, such as social distancing or isolation, can have on the care of millions of patients with non-communicable diseases, who may be indirectly affected, have not been taken into consideration so much.

**Conclusions:**

When dealing with COronaVIrus Disease 19, policy makers and healthcare personnel should consider the indirect effects on the treatment of non-communicable diseases.

## Background

After its outbreak in China, the novel COronaVIrus Disease 19 (COVID-19) [1] spread across the globe with more than 15,000,000 confirmed cases in 188 countries and territories up to 23 July 2020 [2]. Many lessons can be learnt from what has been experienced over the past months. When COVID-19 cases sharply increase, strict infection prevention and control (IPC) strategies are implemented. Two paradigms, containment and mitigation, are usually taken into consideration [3]. Containment is designed to prevent community transmission at the start of an outbreak, by tracking disease dissemination to allow for targeted quarantines. Mitigation strategies are implemented when disease outpaces containment, by promoting travel restrictions, closing schools, canceling sporting events, or even the blockade of productive and commercial activities. The impact of IPC measures is likely to depend on how early they are taken, in the context of local epidemiological, social, and political situations [4, 5]. However, at an early stage, decision makers are often afraid of the economic consequences, and conflicting messages may be released to the population [6–8]. When, finally, COVID-19 cases rapidly increase in a few days or weeks [2, 9, 10], emergency health facilities may be overwhelmed [8, 11, 12], and decision makers may not have enough time to accept, adapt, and implement their response accordingly [13]. Resources are then mainly allocated to enhance emergency care, and are deflected from facilities for non-communicable diseases (NCDs) (including cardiovascular diseases, cancer, chronic kidney disease, chronic liver disease, chronic respiratory disease, endocrine and metabolic disorders) [14]. For their part, patients with NCDs are reluctant to visit health facilities for fear of becoming infected, and new care models, including telemedicine, may not yet be fully implemented. The diffidence of NCD patients in seeking assistance and the inability of healthcare providers to assist them can become major challenges. If we consider the hundreds of millions of patients with NCDs, the impact of COVID-19 on NCD-related deaths (in uninfected patients) is likely to become more significant in the coming months than those directly affected by the infection.

In the upcoming sections, we will briefly summarize the epidemiological trajectory of COVID-19 impact on the world, highlighting the lessons learnt in some countries in different world areas, bringing to the forefront the implications in caring for patients with NCDs affected indirectly. China and South Korea soon passed the peak of the epidemic; Italy, Spain, France, Germany, and other European countries are exiting this phase; the United States (U.S.), South America, and Africa are now experiencing an upward trend while Australia and New Zealand have largely contained the infections. Considering that many low-income regions have not yet gone through the most severe phase, lessons learnt could be effectively applied to establish new outpatient care models based on the successes in other regions, before COVID-19 becomes an unsolvable crisis that could cripple many fragile health systems [15].

## Health policy and lessons learnt

**Fig. 1:**
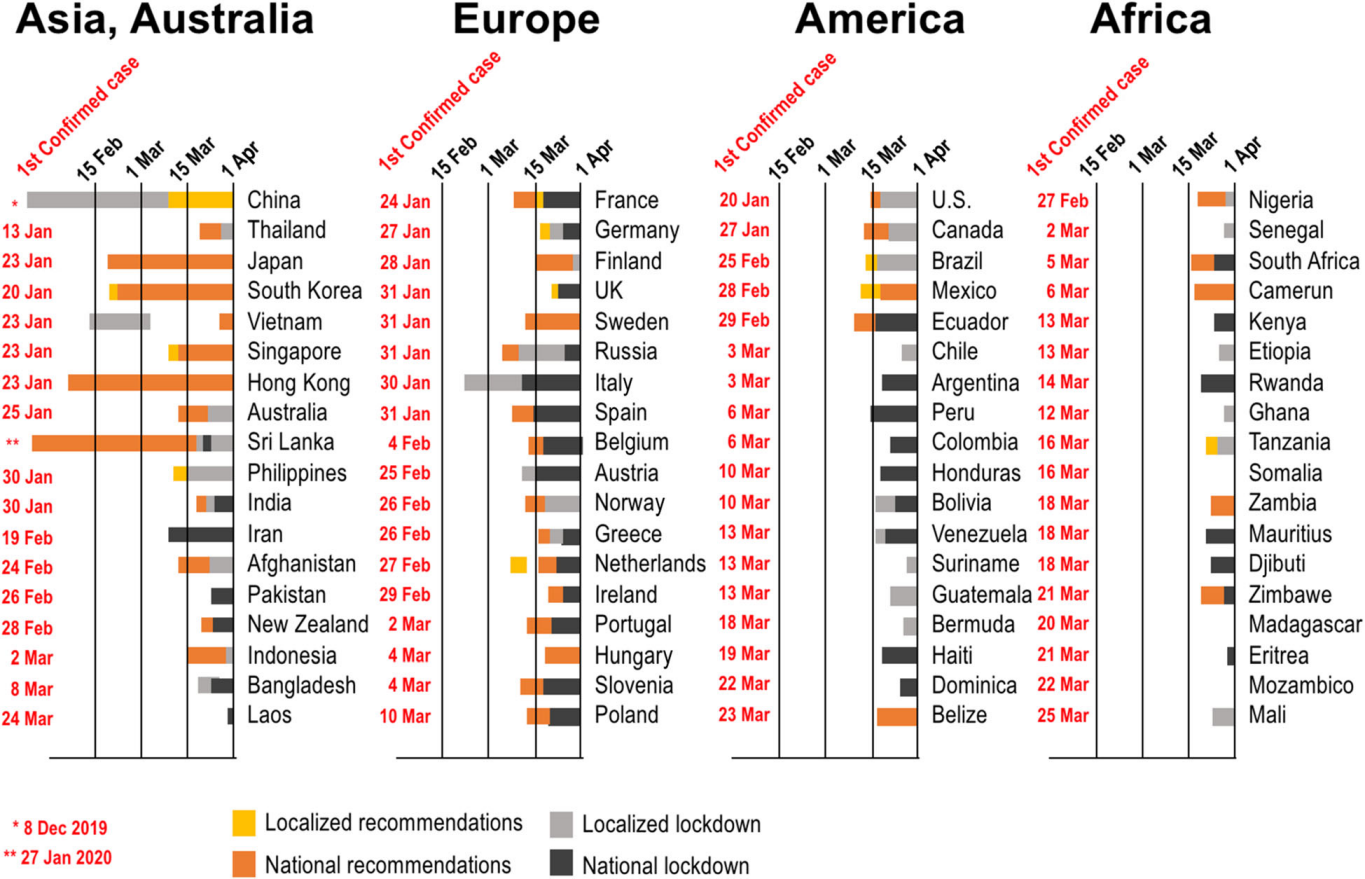
Relationship between the first confirmed case of COVID-19 and establishment of restriction measures in countries of different world areas

Most countries moved from containment to mitigation, albeit at differing paces (Fig. 1). Comparisons and measurements of the effectiveness of interventions are difficult. Although absolute numbers of cases are available [2], the criteria by which they were collected and in particular the criteria for access to diagnosis (what defines a suspected case) are different in different countries. Therefore, there is an uneven and incorrect use of statistics in the comparison of rates between countries. Differences may be related to differences in the definition of “suspected case” (very symptomatic, mild symptomatic, asymptomatic) and thus access to diagnosis rather than to the country’s restriction measures. The World Health Organization (WHO) has been very active in helping all countries across the globe, and it provided a guidance for responding to community transmission of COVID-19 [14, 16, 17]. These indications are largely based on evidence from the analysis of the responses of countries affected by the COVID-19 outbreak early on.

### The birth of two models in Asia

#### China and lockdown

The first COVID-19 outbreak occurred in Wuhan during the “Spring Festival Travel Season” (Chunyun, 10 January 2020) when massive population movement took place. In Wuhan, vigorous and multifaceted measures of containment, mitigation, and suppression were temporally associated with improved control of the epidemic when there was neither an effective drug nor vaccine. In a city with 10 million residents, mitigation measures, such as traffic restriction, cancelation of social gatherings, and home quarantine, were associated with a reduction in the degree of transmission. Between 23 January and 1 February, the local government first blocked all outbound transportation from the city and subsequently suspended public transit and banned all vehicular traffic within the city [18]. When the Chinese government decided to close the city of Wuhan (11 million people), the world was stunned and experts were skeptical. Such a large lockdown, subsequently expanded to almost the entire province of Hubei (60 million people), had never been enforced in the modern world and might not have worked. But today the Beijing approach seems justified [18]. The main mitigation actions undertaken by China were (a) travel restrictions (public and private), (b) closing schools (children at home), and (c) remote work (adults at home).

- Travel restrictions. According to modeling studies [19, 20], Wuhan travel restrictions delayed epidemic progression within China by 3 to 5 days and international travel restrictions did help to slow spread elsewhere in the world until mid-February [19]. Hand washing, self-isolation, and household quarantine were probably more effective than travel restrictions at mitigating this pandemic [19].
- Virtual classroom (children at home). The Chinese Ministry of Education shared instructions for conducting online teaching for students [21], providing 24,000 online courses from over 20 online platforms at no cost [22]. The “home education” program with live streaming lessons has been accepted by more than 20 provinces and is currently followed by over 10,000 primary and secondary schools and 5 million students. According to the Monitoring Report of the United Nations Educational, Scientific and Cultural Organisation (UNESCO) [23], the example was followed by 192 countries who had implemented nationwide closures, affecting about 99% of the world’s student population [20].
- Remote working (adults at home). Since 3 February, when local governments and companies across the nation encouraged workers to stay at home, millions of Chinese have started experiencing the home office for the first time.

The Chinese population proved to be prepared in technological terms thanks to a network of information disseminated on a capillary level in real time. WeChat—a super app that combines messaging, file transfer, video-conferencing capabilities, e-payment, and other functions—is ubiquitous, with over a billion users in China. WeChat and Baidu Maps also released clinical information covering over 100 cities across China and over 3000 clinics. Patients could identify the hospitals designated to treat coronavirus on their phones, dramatically reducing confusion and waiting time.

#### South Korea and tracking

Tracking patients and a broad testing strategy were major contributors in overcoming the COVID-19 outbreak in South Korea [24, 25]. Cell phone GPS data (available to the police authorities), credit card archives (kept by financial institutions), recordings of surveillance cameras, and archives of access to clinics and pharmacies were linked by the government [25]. When an individual is admitted with COVID-19 symptoms, the government retrieves the data spanning the previous 14 days in order to identify people with whom they came into contact (with the help of facial recognition and other technology implemented in public and private areas). Those people potentially exposed to the infection are then contacted and targeted quarantine measures established; these measures would be questionable for privacy violations in many other countries. However, in South Korea, the legislative system was already prepared in this sense by the previous wave of severe acute respiratory syndrome (SARS). Tracking patients, also applied in Hong Kong and Singapore, allowed the countries not to enforce lockdown, limiting quarantine only to those who had close contact with the infected person. In South Korea, an app called “Corona 100m” was also made available for download on South Korean smartphones. The app allows citizens to know if they breach a 100-m (328-ft) radius of the latest tracked whereabouts of a coronavirus patient.

The second cornerstone of the South Korean containment strategy was a broad implementation of finding and immediate testing and isolation [26]. The WHO recommends testing in suspected cases [27]. However, the decision as to what defines a suspected case is left to the country in question. Some countries limited the testing only to very symptomatic cases as China did (e.g., Italy, Spain, and U.S.), whereas others (e.g., Germany) followed the South Korean broad testing strategy. The broad testing strategy was more likely to identify even milder cases [28]. With this approach, South Korea achieved the highest diagnostic rate for COVID-19 [24, 25].

### Lessons learnt in Europe

**Fig. 2:**
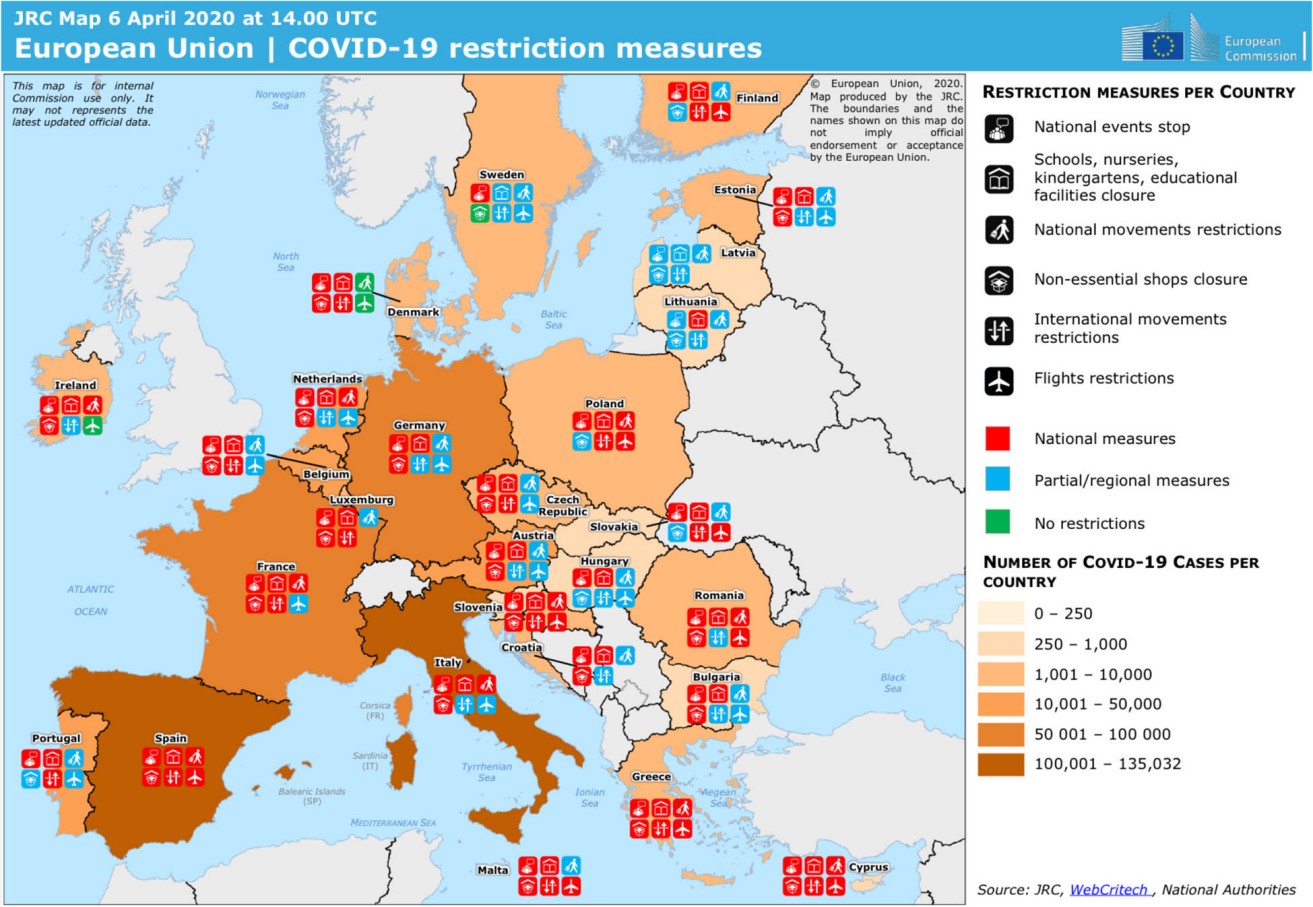
COVID-19 restriction measures in the European Union (reproduced from: https://covid-statistics.jrc.ec.europa.eu/Home/Maps. Licensed under the Creative Commons Attribution 4.0 International (CC BY 4.0) licence)

The two models have been adapted differently to the needs of individual countries in Europe (Fig. 2). However, the UK, Italy, Spain, and France have all exceeded China’s official death toll. All of these nations and the rest of the European Union (EU) now face the difficult question of how and when to end their respective lockdowns. It is not encouraging to see the delays with which this emergency has been understood.

#### Italy

In two cluster zones of Italy, the disease outpaced containment, and mitigation strategies were therefore established. Following the Chinese model, Italy enforced a lockdown limited to a red zone (the extent of which was discussed with the local authorities). However, differently from China, lockdown was then extended at a national level (the largest lockdown in the history of Europe) with different restriction measures (Fig. 2) [29]. Furthermore, differently from China, public transport was never shut down completely and a pass system allowed Italians to move within regional borders depending on need.

Testing strategy was decided at a regional level due to the decentralization of the health system in Italy. The majority of positive cases traced back to two clusters in two neighboring Italian regions, Lombardy and Veneto. Veneto controlled the outbreak within its borders by early and successful implementation of extensive testing, proactive tracing, emphasis on home diagnosis, and primary care. The town of Vó, in Veneto, quashed an outbreak after relentless testing. Other regions in Italy have not replicated the successful model implemented in Veneto. In particular, Lombardy was much less aggressive on these fronts and hospitals in Lombardy were overwhelmed, while Veneto’s hospitals have been spared in comparison.

During the acute phase of the epidemic, most hospitals in Italy converted their facilities into models focused on emergency care while waiting for the arrival of COVID-19 cases. The number of intensive care beds was therefore increased whereas the access to outpatient facilities intended for NCDs was limited.

#### Spain

In Spain, the availability of tests was also limited and disease outpaced containment very quickly. Large public gatherings were still permitted, and the day before the announcement of the cancelation of classes in the Autonomous community of Madrid at all educational levels due to the great increase in cases in the region, 120,000 people gathered in the capital for International Women’s Day (8 March). A few days later, the Spanish government closed all schools and universities, shops, bars and restaurants, and any place open to the public, at a national level. However, citizens were still allowed to go to work and to treatment centers or to purchase basic necessities. All public and private healthcare facilities were placed under the direct control of the Regional public health authorities, the equivalent of the Italian Regions.

The emergency experienced by Spain highlighted the risks of infection for healthcare workers. On 23 March, 5400 (nearly 14%) of Spain’s 40,000 confirmed cases were healthcare professionals. When confirmed, they were sent home, further straining the hospitals [30]. The risks for healthcare personnel are mainly attributed to reduced availability of masks and other Personal Protective Equipment (PPE) (gloves, eye glasses, protective clothing, gowns and head covers, washing paraphernalia). However, the prevalence of infected healthcare professionals is influenced by the testing strategy which in Spain was reserved to symptomatic subjects [30]. In two Dutch hospitals, 6% of symptomatic (fever or respiratory symptoms) healthcare workers [31] were also found to be infected. Conversely, among a non-selected sample of healthcare workers in China, where the lack of PPE was also evident, 110 out of 9684 health workers tested positive, with an infection rate of 1.1% [32]. In the Chinese study, 84.5% of affected health workers had mild or moderate illness. PPE is essential equipment that health workers should be adequately provided with. However, the high prevalence of mild clinical presentation, frequently not including fever, suggests that the current recommended case definition for suspected COVID-19 should be used less stringently, especially for the healthcare workforce [33].

To prevent the possibility that healthcare workers may become prime vectors of transmission, in Wuhan, healthcare workers seeing at-risk patients were housed away from their families [26]. This issue is applicable to all regions because the impact of infection in the healthcare workforce may well be great in low-middle-income countries (LMICs) where human resources to replace them are limited [27].

#### France

In France, a national lockdown was established on 18 March. On 12 March, the situation was declared to be worrying enough to close all schools and universities from 16 March. Yet on 15 March, the same government had urged the French to vote in the first round of the municipal elections in the country’s 35,000 cities, towns, and villages. A second and final round of voting, initially due to take place on 22 March, was then postponed.

In France, the reduction of outpatient facilities undertaken by the healthcare system during lockdown was mitigated by an existing telemedicine service subject to reimbursement by national Social Security since 2018 [34]. Until early March, less than 10,000 teleconsultations a week were invoiced to the National Health Insurance [34]; however, national confinement has skyrocketed service. On the second week of national confinement, 486,369 teleconsultations were performed in a single, although large, national public academic hospital [35]. In the same period, around 44% of general practitioners conducted at least one teleconsultation. The pre-existing telemedicine regulations thus enabled primary care and hospital doctors to switch from scheduled face-to-face consultations with patients they knew personally, to reimbursed teleconsultations, when suitable.

#### Germany

This country, heavily hit by the COVID-19 pandemic, has shown one of the lowest death rates of COVID-19, 0.5% compared to France’s 5.2% and Spain’s 7%. According to data from the European Health Information Gateway [36], Germany has 621 critical care beds per 100,000 people, Italy 275, and Spain 293 [36]. The massive testing strategy implemented by Germany, with more than 500,000 tests every week, played a crucial role in the efficacy of containment. As noted above, the more widely a country tests, the more milder cases it will find. Importantly, Germany does not have a public health laboratory that would restrict other laboratories from performing tests, and thus, the market has been open from the beginning.

On 22 March, the German government also announced lockdown and national curfew with strict social distancing measures. Germany has now started reopening some schools and has allowed businesses and religious venues to open their doors. It also reopened its borders to neighboring countries by 15 June.

#### Russia

The rapid spread of the epidemic in Russia has highlighted the strengths and weaknesses of an extensive, albeit outdated, public health system. Testing capacity is vast and was scaled up quickly (more than 200 laboratories were providing same-day test results). Large testing programs may at least partly explain both the high number of cases and the relatively low mortality rate [37]. However, the COVID-19 outbreak in Russia has been slow but unstoppable. Besides extensive testing, early prevention measures included restricting the border with China. International flights were not limited, and the first confirmed cases in Moscow and in Saint Petersburg were reported to be linked to Italy. Flights to and from Italy, Germany, France, and Spain were then limited. Although the containment and prevention model seemed to have worked, at least initially, the country has gone through just 2 months from having a very low number of infections to becoming the second most important epicenter of the pandemic behind the United States. The quality of health care differs greatly across Russia’s many regions. Russian health workers were reported to be 16 times more likely to die from COVID-19 than their counterparts in other countries [38]. Like in many other countries, there have been conspicuous shortages of PPE.

No emergency situation was officially implemented in Russia, i.e., all the institutional frameworks of power have continued to function during the pandemic. Nevertheless, a presidential decree on the 2nd of April gave governors the power to impose restrictions to prevent the spread of coronavirus, to suspend the activities of enterprises and organizations, and to establish a special procedure for the movement of people and vehicles—except for “vehicles carrying out interregional transportation.”

### The landing in North America

#### The United States of America

On 31 January 2020 [39], 2 weeks after the notification of the first COVID-19 case [40, 41], Health and Human Services Secretary Azar declared a public health emergency effective 27 January 2020, affording the Centers for Medicare & Medicaid Services (CMS) the flexibility to quickly support Medicare beneficiaries (Table 1). At the onset of the outbreak, testing capacity was insufficient and public health officials could not contain the spread of the disease. In particular, aside from the Center for Disease Control (CDC), only diagnostic test makers were initially allowed to develop tests for the coronavirus, while public health laboratories were not. On 29 February, the Food and Drug Administration (FDA) changed its policy and allowed public health agencies, hospitals, and private companies to develop their own assays and perform testing [42]. The country’s testing capacity then substantially increased (running about 50,000 to 70,000 coronavirus tests per day), and currently, there are 70 FDA-authorized tests on the market [43]. However, some limitations still remain: many of these tests are run on manual or semi-automated systems; test makers also face shortages of reagents, swabs, and various collection devices; a patient has to be completely symptomatic to get a test according to guidelines stipulated with Departments of Health. On 4 March, the CDC relaxed the criteria to allow doctors the discretion to decide who would be eligible for tests. More precisely, the criteria for evaluation of persons for testing for COVID-19 were expanded to include a wider group of symptomatic patients.

**Table 1:** The extent of the spread of the virus and timeline of National responses

Country	Timeline	Population	COVID-19 cases (18 June 2020)	COVID-19 deaths (18 June 2020)
**Asia**				
**China**	**27 December** 2019, first notification to the local center for disease control and prevention (CDC) and health commissions; **end of December** 2019, suspension of passenger trains to and from Wuhan; **1 January**, seafood market closure; **20 January**, the NHC started publishing daily data on confirmed and suspected cases; **21 January**, government officials warned against hiding the disease; **22 January**, Hubei announced a Class 2 Response to Public Health Emergency; **23 January**, Wuhan declared lockdown and the province of Zhejiang announced Class 1 Response to Public Health Emergency; **26 January**, the State Council extended the 2020 Spring Festival holiday; 27 January, MoE advised all higher education institutions to postpone the new spring semester; **3 February**, local governments and companies across the nation encouraged remote working (home office); **7 February**, the Chinese Ministry of Education shared instructions for conducting online teaching for students; **7 April**, the end of Wuhan lockdown was declared	1,439,323,776	84,458	4638
**South Korea**	**20 January**, first notification; **4 February**, denying entry to foreigners traveling from Hubei Province; **18 February**, most universities in South Korea postponed the start of the spring semester; **23 February**, all kindergartens, elementary schools, middle schools, and high schools were announced to delay the semester start; **25 February**, Daegu officials were aggressively warning residents to take precautions, while allowing private businesses such as restaurants to stay open	51,269,185	12,257	280
**Europe**				
**Italy**	**23 January**, first notification; **31 January**, flights to and from China suspended and state of emergency declared; **20 February**, patient tested positive in Lombardy; **21 February**, two people tested positive in Veneto; **22 February**, lockdown of 11 municipalities in Lombardy and Veneto (red zone); **1 March**, the Italian national territory was divided into three areas (red zone with whole population in quarantine; yellow zone, social and sport events suspended and schools, theaters, clubs and cinemas are closed; rest of the national territory, where safety and prevention measures are advertised in public places); **4 March**, shutdown of all schools and universities nationwide; **8 March**, lockdown to all of Lombardy and 14 other northern provinces and on the following day to all of Italy; **11 March**, all commercial activities except for supermarkets and pharmacies were closed; **21 March**, the Italian government closed all non-essential businesses and industries and restricted movement of people; **26 April**, movements across regions are still forbidden, while the ones between municipalities are allowed only for work and health reasons, as well as for visits to relatives; reopening of manufacturing industries and construction sites is allowed; **18 May**, most businesses could reopen, and free movement was granted to all citizens within their Region	60,465,149	237,828	34,448
**Spain**	**31 January**, first notification; **14 March**, lockdown was imposed; **17 March**, a national state of emergency was declared, mobilizing the army and ordering the closure of schools and universities, shops, bars and restaurants, and any place open to the public. Citizens’ movements are allowed only to go to work and to treatment centers or the doctor or to purchase basic necessities, such as food and medicine. All the hospitals and private healthcare facilities in the country were placed under the direct control of the regional public health authorities	46,754,778	244,683	27,136
**France**	**24 January**, first notification; **16 March**, the beginning of a lockdown period was announced and all schools and all universities were closed; **17 March**, ban on all travel except relating to professional activity, buying essential goods, health or family reasons or brief individual exercise, closure of all non-essential public places, including restaurants, cafés, cinemas, and nightclubs; **17 March**, the Direction générale de la Santé (DGS) asked Santé publique France to buy urgently 1.1 million of FFP2 masks; **11 May–1 June**, phase 1 of lockdown lifting; **2 June**, phase 2 of lockdown lifting	65,267,844	194,805	29,578
**Germany**	**27 January**, first notification; **13 March**, school and kindergarten closures, postponing academic semesters and prohibiting visits to nursing homes to protect the elderly; **15 March**, borders to five neighboring countries were closed; **22 March**, prohibited physical contact with more than one person who resides outside of a household, curfews were imposed in six German states while other states instead opting for strict social distancing measures	83,783,942	188,604	8868
**UK**	**31 January**, first notification; **3 March**, the UK Government unveiled their Coronavirus Action Plan; **12 March**, the government announced it was moving out of the contain phase and into the delay phase of the response to the coronavirus outbreak; **17 March**, NHS England announced that all non-urgent operations in England would be postponed from 15 April to free up 30,000 beds; **26 March**, the Health Protection (Coronavirus, Restrictions) (England) Regulations 2020 made the sweeping restrictions (national lockdown) legally enforceable; **4 April**, Johnson was admitted to Hospital, **12 April**, left the hospital; **April**, shortage of chemical reagents needed for COVID-19 testing; **10 May**, Prime Minister Johnson asked those who could not work from home to go to work, avoiding public transport if possible	67,886,011	300,717	42,238
**Russia**	**30 January**, Chinese–Russian border was shut; **31 January**, first notification; **17 March**, closing all cultural institutions under its jurisdiction, including museums, theaters, symphonies, and circuses; **23 March**, all Russian schools were closed; **24 March**, instruction to regional authorities to suspend activities of any nightclubs, cinemas, and children’s entertainment centers and to ban hookah smoking at any restaurants or cafes; **25 March**, the 2020 Russian constitutional referendum was postponed; **27 March**, international flights were grounded; **28 March**, all universities were closed; **30 March**, lockdown started; **11 April**, Moscow’s mayor introduced a digital pass system to enforce the coronavirus lockdown; **11 May**, end of the national non-working period	145,934,462	552,549	7468
**Middle East and North Africa (MENA)**				
**Israel**	**21 February**, first notification; **11 March**, Israel limited gatherings to 100 people; **12 March**, all universities and schools were closed; **17 March**, a Knesset committee approved the contact-tracing program	8,655,535	19,894	303
**Iran**	**24 February**, first notification; **25 February**, declared lockdown procedures	83,992,949	195,051	9185
**Tunisia**	**2 March**, first notification; **12 March**, schools and all universities were closed; **18 March**, curfew from 6 p.m. to 6 a.m.; **23 March**, general confinement	11,818,619	1128	50
**Egypt**	**14 February**, first notification; **19 March**, restaurants, cafes, nightclubs, and public places throughout the country were closed from seven in the evening until six in the morning, decision to close the airports and suspended all air travel; **21 March**, decision was made to suspend prayers in all of Egypt’s mosque, the Coptic Orthodox Church also announced the closure of all churches and the suspension of ritual services, masses, and activities	102,334,404	49,219	1850
**Yemen**	**10 April**, first notification	29,825,964	902	244
**Africa**				
**Nigeria**	**27 February**, first notification; **19 March**, Federal government announced the closure of tertiary institutions, secondary and primary schools; Anambra State government announced the closure of their schools and suspension of public gatherings indefinitely, tertiary institutions to close from 20 March, while primary and secondary schools to close from 27 March, Ogun State government extended an earlier ban to schools and religious centers in the state indefinitely; **21 March**, Nigeria announced the closure of their international airports, Enugu, Port Harcourt, and Kano airports; **1 June**, the federal government announced the reopening of domestic airline operations from 21 June and shortened the curfew from 10 p.m. to 4 a.m.	206,139,589	17,735	469
**South Africa**	**5 March**, first confirmed case; **15 March**, declaration of a national state of disaster; **26 March**, national lockdown started; **1 May**, a gradual and phased easing of the lockdown restrictions started, lowering the national alert level to 4, to be lowered to level 3 from 1 June	59,308,690	80,412	1674
**Mozambique**	**22 March**, first notification	31,255,435	651	4
**America**				
**U.S.**	**20 January**, first notification; **29 January**, the White House Coronavirus Task Force was established; **31 January**, public health emergency was declared; **2 February**, prevent the entry of most foreign nationals who had recently traveled to China; **29 February**, the Food and Drug Administration (FDA) began allowing public health agencies, hospitals, and private companies to develop tests and perform testing; **5 March**, the CDC relax the criteria to allow doctors discretion to decide who would be eligible for tests; **13 March**, national emergency was declared; State and local responses to the outbreak were different including prohibitions and cancelation of large-scale gatherings (including festivals and sporting events), stay-at-home orders, and the closure of schools; **19 March**, the State Department suspended routine visa services at all American embassies and consulates worldwide	331,002,651	2,163,290	117,717
**Brazil**	**28 January**, the Ministry of Health in Brazil raised the emergency alert to level 2 of 3, considering an “imminent threat” for Brazil; **3 February**, the Minister of Health declared a Public Health Emergency of International Concern; **25 February**, first notification in Brazil; **21 March**, the State of São Paulo declared a state-wide quarantine; **7 May**, cities in the northern states of Amazonas and Pará begun issuing lockdown measures; **9 May**, the government of Rio Grande do Sul established a new social distancing plan.	212,559,417	955,377	46,510
**Ecuador**	**29 February**, first notification; **14 March**, the government announced the closure of its borders; **24 March**, police moved to dismantle open markets; **early April**, the health system in Guayas Province was overwhelmed	17,643,054		

The effect of the highly decentralized U.S. health system, a similar situation to Italy, must also be considered. Measures were imposed at a local community level with different scope and severity. Communities generally implemented the so-called social distancing measures as well, such as canceling conferences, sporting events, and other large gatherings. This also happened for schools. The CDC advised colleges to suspend classes and events only if they had identified cases of COVID-19 within their communities. With a proactive action, about 300 universities and colleges around the U.S. canceled in-person classes in March and 520 campuses across 47 states shut down [44]. Accordingly, different strategies were implemented across different U.S. states and cities, with clearly different results. This makes the U.S. a much closer comparison to Italy, rather than to Asian countries such as China and South Korea.

### Latin America

Most countries in the region, including Chile, Argentina, Colombia, and Brazil, have implemented stay-at-home measures, shut down borders, and closed businesses (Fig. 1) [45]. Much hinges on how authorities manage the current health crisis and the economic fallout in countries with fragile health systems, a high proportion of workers in the informal sector, and weak fiscal and monetary firepower.

#### Brazil

In Brazil, the Ministry of Health raised the emergency alert and declared a Public Health Emergency of International Concern even before the first case in the country (Table 1). However, few country-wide measures to slow the spread of the virus were taken and the country faces some of the highest numbers of infection in the world. Since the federal government decided not to cancel classes in the whole country, municipal, state, and private schools and universities had different reactions regarding suspension, replacement with remote education, or simply postponement. In Brazil, there have been no nationwide guidelines for primary healthcare services in the COVID-19 response. Brazil performed testing only on patients with severe symptoms, and a shortage of materials such as masks, N95 masks, gloves, and hand sanitizer was reported [46, 47]. Since community health workers in Brazil are not considered to be health professionals, only an estimated 9% have received infection control training and personal protective equipment [46].

#### Ecuador

In Ecuador, described as a possible epicenter of COVID-19 in Latin America, minor measures were taken and the health system was overwhelmed in Guayas Province [48]. The number of deaths is believed to be significantly higher than the official figure due to a low rate of testing [48].

### Eastern Mediterranean region

As of 6 May, the disease has spread across the 22 countries in the Eastern Mediterranean region. Available figures (Table 1) probably underestimate the extent of the spread of the virus because of the inadequate case reporting across the region. The Eastern Mediterranean region is home to both the wealthiest and the poorest countries in the world, and the responses of countries were uneven, ranging from restrictive temporary lockdowns to denial and more lax approaches [49]. Iran reported the first confirmed cases of infection in the region. The government canceled public events and Friday prayers, closed schools, universities, shopping centers, bazaars, and holy shrines, and banned festival celebrations. Plans to quarantine entire cities and areas were initially rejected by the government, and heavy traffic between cities continued.

The Israeli government proposed allowing the Israel Security Agency to follow the South Korean model by tracking the previous movements of people diagnosed with coronavirus through their mobile phones to identify people with whom infected individuals came into contact. The Knesset committee approved the contact-tracing program. Within the first 2 days, 400 individuals who had been in proximity to an infected person were advised to enter a 14-day self-quarantine period. The security measure was in place for only 30 days. Critics branded the proposal an invasion of privacy and civil liberties.

### The African Region

The COVID-19 pandemic continues to expand in the African Region, and all African countries now have confirmed cases [50, 51]. According to the official bulletin of the African Union Disease Control and Prevention Centers (CDC Africa), South Africa remains the country with the highest number of cases ascertained. Delayed and incomplete data reporting, lack of data sharing, and unavailability of testing may limit epidemiological analyses. At the beginning of February, only Senegal and South Africa had the ability to test for the novel coronavirus. The WHO has been supporting African governments considerably, and 42 laboratories in capital cities are now performing testing [52]. The WHO is also helping local authorities craft radio messaging and TV advertisements to inform the public about the risks of COVID-19 and what measures should be taken [50–52]. Countries such as South Africa (with the highest burden of HIV) has acted quickly with severe social distancing or isolation restrictions imposed [53]. Kenya, Ghana, Rwanda, Mali, and Nigeria are among other African countries to impose restrictions to curb transmission of the coronavirus [54]. Several African countries have banned weddings, funerals, and large religious gatherings. Many countries have also shut down schools [55]. In most LMICs including many African countries and several South Asian countries, the health system is weak, especially for specialized care such as intensive care. Creative approaches are required to protect people from contamination, such as strict social distancing policies and intensification of contact tracing; these are especially necessary given the strong social structures such as the extended family systems in many of these countries.

## Impact of the COVID-19 pandemic on NCDs

The negative implications of IPC strategies on cardiovascular health have recently been discussed [56, 57]. In China, a significant reduction in emergency department (ED) visits related to different disciplines was observed early on in the first weeks of the pandemic [58]. Although this may highlight the overuse of EDs by non-emergency and non-complex cases that could be managed by general practitioners, there may also be a worrisome tendency to postpone consultations with specialists, even when necessary [59]. The consensus is that patients were avoiding going to hospitals because they feared getting infected with COVID-19 [60]; patients with NCDs might indeed consider themselves at increased risk of severe COVID-19 [61–68] and therefore avoid or delay referral.

According to a recent report of the CDC, the total number of U.S. ED visits was 42% lower than during the same period a year earlier [69]. The largest declines were in visits for abdominal pain and other digestive or abdominal signs and symptoms and essential hypertension [69]. Although not in the top 20 declining diagnoses, visits for acute myocardial infarction also decreased. Recent reports from scientific societies in Italy [70, 71], Spain [72], and the U.S. [73] also suggest a substantial reduction in admission for stroke or myocardial infarction since the start of the outbreak. Importantly, people with heart attacks who arrived at the hospital did so late. Health messages that reinforce the importance of immediately seeking care for symptoms of serious conditions, such as myocardial infarction, are necessary.

Another important issue is a shortage of healthcare staff and facilities to cover both COVID-19-related illness and all other routine medical care. A nationwide survey conducted in April found that a quarter of cancer patients receiving active treatment in the U.S. had seen their care delayed. A recent WHO survey looked at the extent of the disruption of services for the prevention and treatment of NCDs [74]. The survey showed that 81% of responding countries abolished rehabilitation care, and 47% completely or partially abolished palliative care facilities. Additionally, while two thirds (63%) reported that services for cardiovascular disease emergencies have not been disrupted, this was only the case for a quarter (24%) of hypertension management services. In particular, admissions to facility care perceived as non-essential were limited, and population screening programs were temporarily suspended in 61% of cases [74].

On the other hand, many countries have realized the impact of the COVID-19 pandemic on NCD patients and experimented new care models [75–85].

### Managing NCD outpatients in the COVID-19 era

Simple remote approaches, such as telephone consultation, are already key to many NCD management programs, such as for heart failure [75, 76], and can now be adapted to limit the need for a clinic visit to receive drug prescriptions. In this context, telemedicine [77, 78], particularly video consultations [79], has been promoted and scaled up to reduce the risk of transmission, especially in France [34], the UK [79], and the U.S. [80, 81]. In France where telemedicine was already authorized, reimbursed, and actively promoted, the pre-existing regulation has recently been reinforced [82] to limit the number of individuals grouping in waiting rooms, to screen and detect suspected patients, and to allow follow-up from home.

Initiatives have been taken for authorization and reimbursement of teleconsultation in the U.S. [83] and various European countries since the outbreak of COVID-19 [84, 85]. Belgian public health insurance recently approved the reimbursement of teleconsultations by doctors [86]. Belgium is in line with many other countries worldwide, such as Israel [87], France [88], Italy [89], and Australia [85], which all use telemedicine to a growing extent to fight the COVID-19 pandemic.

Most countries, however, lack a regulatory framework to authorize, integrate, and reimburse telemedicine services, including in emergency and outbreak situations [80, 90]. Two possibilities are currently available for patients: [1] direct-to-consumer telemedicine with private providers mostly relying on out-of-pocket or private insurance payment and [2] free solutions, mainly from companies like WhatsApp, Skype, or Facetime, that may not respect national health data privacy and security requirements. Although these solutions may be useful to support and alleviate the pressure on healthcare systems during the outbreak, to date, they are mostly not integrated within national healthcare systems and do not share data with public health authorities for epidemiological surveillance [34]. For countries without integrated telemedicine in their national healthcare system, the COVID-19 pandemic is a wake-up call to adopt the necessary regulatory framework to support wide adoption of telemedicine [34]. In this context, Italy does not include telemedicine in the essential levels of care granted to all citizens within the National Health Service.

Freely accessible home-based telemedicine software platforms respecting privacy and security requirements are now available [91]. Telemedicine systems range from simple video conference tools to perform a secure medical consultation (interactive medicine) to sophisticated software and hardware combinations for diagnosing conditions and developing treatment plans (store and forward, and remote patient monitoring). The store and forward type of telemedicine allows providers (primary care physicians) to share patient information with a specialist in another location. Remote patient monitoring permits providers to monitor their patients in their own homes [91, 92]. Doxy.me is a free platform where the physician can create a virtual room and start providing telemedicine services to patients. The software is certified for storing the information relating to patients and treatments provided to them. AMC Health focuses on health monitoring and care coordination via FDA-approved medical devices. This includes tracking and Bluetooth connectivity to allow the operation of remote devices to send biometric data, which can be provided by either the patient or a caregiver. Telemonitoring may add significant value to managing chronic diseases and avoiding infection with COVID-19.

### Problems/difficulties we are still facing today and possible solutions

#### Language and technological barriers

Remote consultation poses challenges for clinicians who need to deliver care to patients with migrant backgrounds or language/technological barriers. WHO leaders appeal for more attention for refugees and non-documented migrants who often have poor language proficiency and legal, administrative, and financial obstacles when accessing health care [93]. Although migration flows are likely to decrease, the number of international migrants may not decrease immediately, as migrants are unable to return to their countries due to travel bans and disruption of transport services. Migrant workers tend to be more vulnerable to loss of jobs and wages in the host country than native workers. In addition, lockdown in work camps and dormitories can increase the risk of contagion among migrant workers. Finally, telemedicine as well as the current mantra “Stay Home” can hardly be extended to homeless populations. Cities with large homeless populations may face unique challenges when trying to contain COVID-19 [94, 95]. Policy makers should consider the risk of increasing health inequality [96].

#### The elderly

As in other health emergencies, institutionalized elderly people are often the invisible part of the crisis; outbreaks in nursing homes are now a major problem [97, 98]. It is important to understand specific transmission dynamics to inform strategies to prevent introduction or spread of COVID-19 in nursing homes for the elderly. COVID-19 is often introduced by the staff, who are much less equipped and educated about the handling of the disease than hospitals are.

Suspension of visits and personal aids with extended barrier measures, the establishment of safe supply chains, isolation of cases, sanitation, and limitation of internal activities were recommended in France to reduce the risk of introduction of severe acute respiratory syndrome-coronavirus-2 (SARS-CoV-2) in retirement facilities. However, in France, as of 15 March 2020, people older than 75 years accounted for 20% of the confirmed cases but 79% of the deaths [99].

The issue of loneliness is especially relevant for the elderly, who may not receive the optimal social support necessary at this stage in life, due to the physical and social distancing guidelines. The elderly living in nursing homes may experience increased anxiety due to reports that some facilities have been severely affected by deaths and spread of infection. The use of online technology is a useful means of providing support networks and a sense of belonging, but clashes with the existence of economic disparity in accessing or reading digital resources. A more sustainable strategy is one that involves simple interventions such as more frequent telephone contacts with family and close friends, voluntary organizations or health professionals, or community outreach projects that provide peer support during forced isolation. Interventions to support mental health may be crucial in these populations.

#### Low-income regions

In low-income regions, people often live in very close proximity to each other (e.g., in Pakistan, the majority of households include more than 6 people). The economic consequences of COVID-19 are likely to be significant, and this will affect NCD patients who rely on their families for their care financially. Often, people living in these settings also have both malnutrition and NCDs. Many countries are advising their citizens to stay at home. Staying at home will directly affect economic activities particularly for the most disadvantaged members in society. With the sudden loss of income or access to social support, vulnerability can change dynamically depending on the policy response [96]. A creative approach can be adopted locally. In most African countries, hypertension clinics are still running and a system of triage by phone would mean a lower level of contamination. WHO recommendations for the rational use of PPE in healthcare and community settings have been provided [100, 101]. However, there may be a shortage of disposable masks and protective clothing; in this case, (non-disposable) clothing and some masks can be sterilized and re-used. We need to propose that all people who go to markets, where there is high population density, and who use public transport, should use PPE to protect themselves and others from contamination. In the absence of masks, people should use something, even a piece of cloth washed every day [102]; according to the CDC recommendations, in settings where facemasks are not available, homemade masks (e.g., bandana, scarf) can be used as a last resort [103]. However, homemade masks are not considered PPE, since their capability to protect is unknown.

#### Clinical trial integrity

The coronavirus pandemic threatens the integrity of ongoing clinical trials for interventions that may benefit NCD patients [104]. Mitigation efforts may interfere with all aspects of a clinical trial. Both the U.S. FDA (18 March) [105] and the European Medicine Agency (25 March) [106, 107] issued guidance for industry, investigators, and institutional review boards conducting clinical trials during the coronavirus (COVID-19) pandemic.

The impact on the data collection, analysis, and interpretation of results for each trial will need a thorough case-by-case assessment. The approach to stop randomized trials that do not provide a clear immediate benefit to the enrolled participants is difficult to apply because the benefits of the interventions are unknown until the completion of the study. Supporting ongoing trials could conversely help millions of people obtain substantial and lasting health benefits that will be important once the coronavirus pandemic ends. Therefore, efforts and resources should be dedicated to supporting randomized trials using creative and thoughtful methods and proactive planning. The adjustment of protocols to limit hospital visits for outpatients may facilitate continuous adherence to the intervention and recruitment for ongoing trials. The conclusion of ongoing trials could lead to important benefits for many patients with NCDs [104].

#### Fake news

Misinformation stating that the use of antihypertensive medication, namely angiotensin-converting enzyme inhibitor (ACEI) and angiotensin-receptor blocker (ARB) treatments, may increase the risk of SARS-CoV-2 infection or the severity of the disease has created confusion for millions of hypertensive patients and clinicians alike. This point is crucial because such a message may dissuade patients from keeping to their treatment regimen. At this stage, there is no clear data on humans from randomized controlled trials, to recommend any clinical action. Clinical societies, the European Society of Hypertension, the European Society of Cardiology Council on Hypertension, and the International Society of Hypertension, have reinforced that there is currently not enough information to make recommendations and have reiterated that there is no reason to stop ACEI/ARB treatment in stable patients [108–111]. The most common fake news has been discussed, and the WHO is launching an official app for iOS and Android that is being designed to combat the spread of misinformation about COVID-19 [112].

## Conclusions

In the initial stages of the COVID-19 pandemic, decision makers and people were often reluctant to enforce IPC strategies due to the financial consequences of an economic shutdown. In many countries, IPC strategies were overlooked and conflicting messages were released to the population.

When IPC strategies were finally implemented, the media and health systems focus on the treatment and outcome of COVID-19 patients with a consequent lower priority for patients affected by chronic NCDs, such as heart disease, diabetes, and cancer. Outpatient facilities for NCDs (mainly hypertension and diabetes) have been suspended in several countries.

While responding to COVID-19, policy makers should consider the risk of increasing health inequalities. Considering that many LMICs have not yet reached the peak of the COVID-19 pandemic, it would be helpful for clinicians to share with their patients the treatment and care strategies to follow during possible future lockdown periods.

## Data Availability

Not applicable.

## Abbreviations

ACEIs: Angiotensin-converting enzyme inhibitors
ARBs: Angiotensin-receptor blockers
CDC: Center for Disease Control
COVID-19: COronaVIrus Disease 19
ED: Emergency department
EU: European Union
FDA: Food and Drug Administration
IPC: Infection prevention and control
LMICs: Low-middle-income countries
NCDs: Non-communicable diseases
PPE: Personal Protective Equipment
SARS: Severe acute respiratory syndrome
SARS-CoV-2: Severe acute respiratory syndrome-coronavirus-2
UNESCO: United Nations Educational, Scientific and Cultural Organisation
U.S.: United States
WHO: World Health Organization
